# Renal Cell Carcinoma in a Horseshoe Kidney Treated with Laparoscopic Partial Nephrectomy

**DOI:** 10.1155/2018/7135180

**Published:** 2018-06-07

**Authors:** Shinji Ohtake, Takashi Kawahara, Go Noguchi, Noboru Nakaigawa, Kimio Chiba, Hiroji Uemura, Masahiro Yao, Kazuhide Makiyama

**Affiliations:** ^1^Department of Urology, Yokohama City University Graduate School of Medicine, Yokohama, Japan; ^2^Department of Urology, Kawasaki Municipal Ida Hospital, Kawasaki, Japan; ^3^Departments of Urology and Renal Transplantation, Yokohama City University Medical Center, Yokohama, Japan; ^4^Department of Urology, Kanagawa Cancer Center, Yokohama, Japan

## Abstract

**Introduction:**

Horseshoe kidney is one of the most common congenital renal fusion anomalies. Due to its poor mobility and abnormal vasculature form, surgeons should pay close attention to all anatomical variations.

**Case Presentation:**

An 83-year-old woman was referred to our hospital because of left renal tumor in a horseshoe kidney incidentally found by her previous hospital. We performed laparoscopic partial nephrectomy. The pathological diagnosis was clear cell renal cell carcinoma. G2 INF*α* V-pT1a with a negative surgical margin. No evidence of recurrence has been noted, and the renal function is well preserved at 28 months after surgery.

**Conclusion:**

When performing laparoscopic partial nephrectomy for renal carcinoma, especially a horseshoe kidney, preoperative imaging is crucial for identifying the location of the renal vessels.

## 1. Introduction

Partial nephrectomy for small renal tumor has the same cancer control rate as nephrectomy and good results in terms of the preservation of the renal function. Laparoscopic partial nephrectomy is now the gold standard for such tumors [1–3].

Due to anatomical issues, surgical planning is difficult when a tumor arises in a horseshoe kidney. Laparoscopic partial nephrectomy for horseshoe kidney tumors therefore remains challenging.

We herein report a case of small renal cell carcinoma arisen in a horseshoe kidney successfully resected by laparoscopic partial nephrectomy using preoperative three-dimensional (3D) imaging.

## 2. Case Presentation

An 83-year-old woman was referred to our department for further examination of a left renal tumor 20 mm in diameter in her horseshoe kidney. She had no remarkable medical history except for uterine cancer at 49 years of age. The laboratory data showed slight anemia and a low liver function (Hb 12.3 g/dL, AST 52 IU/L, ALT44IU/L, and LDH 201 IU/L).

**Figure 1:**
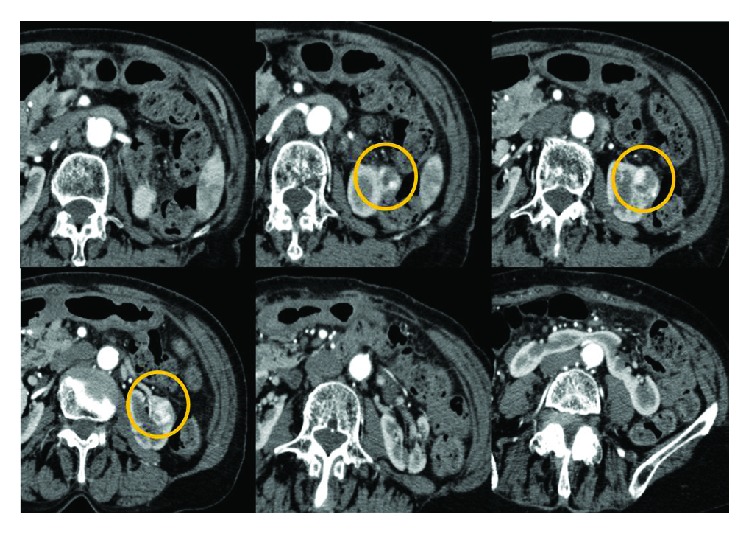
Contrast-enhanced CT. An enhanced tumor 20 mm in diameter was observed in the upper pole of the left-side horseshoe kidney.

**Figure 2:**
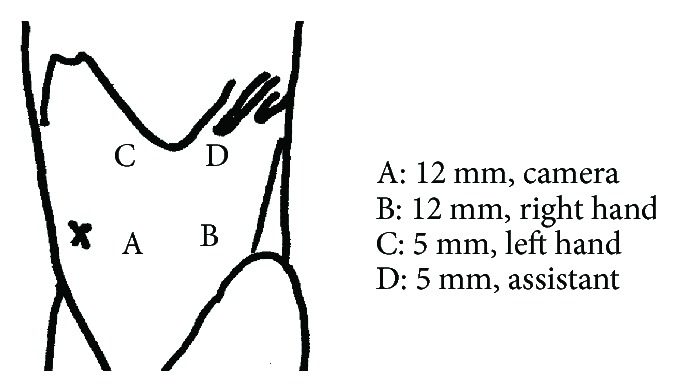
Laparoscopic ports in this case.

**Figure 3:**
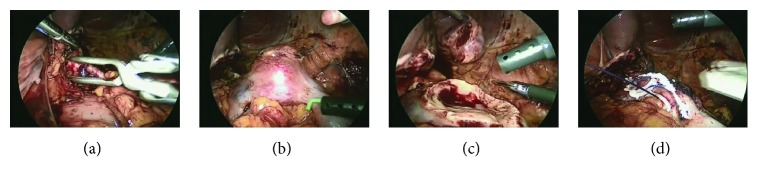
Laparoscopic findings. (a) Left renal artery, (b) tumor, (c) after partial nephrectomy, and (d) after suturing.

Contrast-enhanced computed tomography (CT) showed a renal tumor covered with a capsule and buried by nearly 30%. The tumor was supplied by four arteries toward the left kidney (Figure 1). We set the laparoscopic ports as shown in Figure 2. We first approached intraperitoneally and then cut the peritoneal and approached to the renal helium. We encountered a thin artery and cut it after confirming the supplied area by clamping. We then clamped the main artery and cut the tumor with a 1 mm surgical margin. No urinary tract leakage was observed. After coagulation using bipolar forceps, we sutured using 1-0 Vicryl (Ethicon, Cincinnati, OH, USA) then sprayed with Arista AH (BARD; Warwick, RI, USA). We then fixed the thread as shown in Figure 3. After confirming the lack of active bleeding, we placed a drainage tube and closed the incision. The total operation time was 2 h 39 min, with 11 min of clamping.

A histopathological examination revealed clear cell carcinoma (grade 2, INF*α*, v[−], pT1a). The patient was discharged 11 days after surgery and has been free from recurrence for 43 months.

## 3. Discussion

Horseshoe kidney is a congenital abnormality in 1 of every 400 to 1000 individuals, and the incidence in men is twice compared with that in women. Horseshoe kidney is often seen in patients who have a chromosome disorder, with a rate of 7% in those with Turner syndrome [4]. Due to accompanying anatomical disorders, one-third of patients have renal stones or hydronephrosis due to stricture of the ureteropelvic junction [5, 6]. No association has been noted between horseshoe kidney and renal cell carcinoma, and only 200 cases have been published [7].

**Figure 4:**
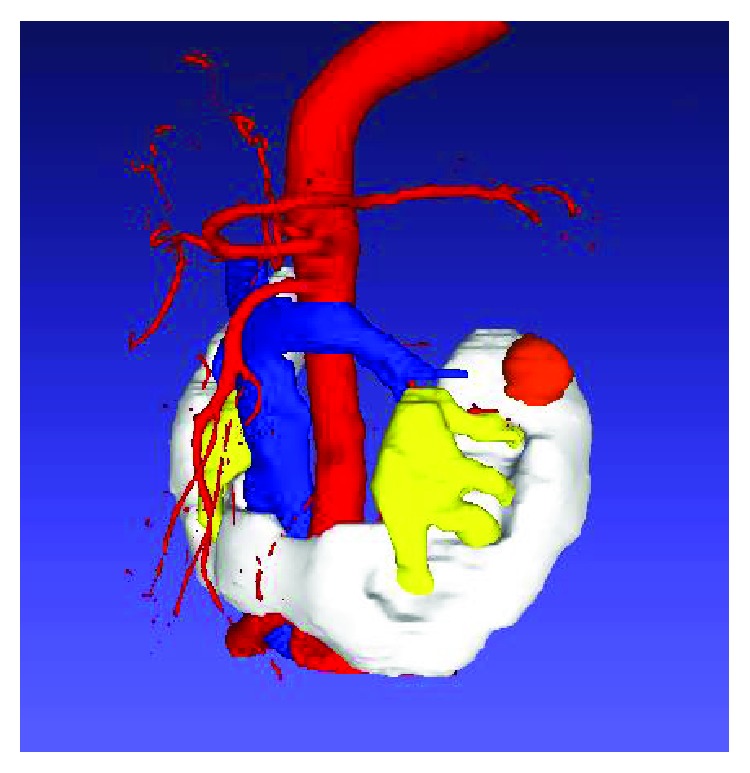
CT image of the tumor in the horseshoe kidney.

Laparoscopic surgery was recently reported in several cases of horseshoe kidney with isthmectomy, pyeloureteroplasty, nephrectomy, and partial nephrectomy [8, 9]. However, given the complicated vessel anatomy and poor mobility, surgical planning is difficult when a tumor arises in a horseshoe kidney. The tumor location was suggested as the most important factor influencing the selection of an intra- or retroperitoneal approach. Molina and Gill showed that an intraperitoneal approach was more appropriate for anterior to external areas, while a retroperitoneal approach was more appropriate for posterior to external areas [10]. In the present case, the tumor was located in the anterior-external area, so an intraperitoneal approach was selected. Using this approach, a large operation area was obtained, which helped reduce the surgical operation time; our suturing technique also helped shorten the operation by reducing the amount of bleeding [11]. We also constructed 3D images (OsiriX, Pixmeo; Bernex, Switzerland) to understand the anatomical structure more easily (Figure 4).

Thus far, four cases of laparoscopic partial nephrectomy for renal tumor in a horseshoe kidney have been reported. In previous cases, the surgical approach was determined by the tumor location. In these reports, 2 cases had renal cancer, and 1 case described in the literature showed no recurrence at 12 months postoperatively [12]. Our case remains free from recurrence at 43 months postoperatively with a preserved renal function.

## 4. Conclusion

We herein reported a case of renal cell carcinoma in a horseshoe kidney successfully treated by laparoscopic partial nephrectomy. Preoperative detailed planning using CT positively contributed to performing successful surgery.
